# Structure and Management of Modern Dwellings

**Published:** 1919-09-27

**Authors:** 


### TENEMENT HOUSES.

### II. Structure and Management of Modern Dwellings.

In the first article we described many of the evils associated with the older type of tenement blocks, and showed that some of the grosser defects at any rate can be remedied by strict supervision and enforcement of ordinary cleanliness. Supervision, however, cannot remedy such structural defects as want of w.c. accommodation or proper facilities for disposal of house refuse, and it is our purpose to discuss in this article some of the essentials in the structure and management of model dwellings. In considering this subject it will be convenient to discuss as it concerns (1) The individual tenement, and (2) The relation of the tenements to one another.

### Self-contained Tenements.

Taking first the individual tenement, it must be *self-contained*, for by this principle you sum up practically the whole requirements for the modern tenement, while avoiding the faults of the older forms. Now what is meant by a tenement being self-contained? The answer is that it must have within its boundary-walls every essential which is properly found in a good-class workman's cottage. These will consist of from one to four bedrooms, according to the size of the family they are intended for, one living-room or kitchen, a scullery, a bathroom, and a w.c. which opens either on the passage, or preferably on to a small open-air space level with and belonging to the tenement. In the case of a one-roomed tenement the principal room may serve as a bed-sitting room, and the other appurtenances will be a scullery, w.c., and bathroom. In the larger tenements a small parlour or sitting-room may be provided, or one of the bedrooms could be used for this purpose, and the additional requirement would then be a kitchen, scullery, w.c. and bathroom. In most of these requirements there will be little difficulty in making your tenement equal to a small cottage, but on one point the cottage has a great advantage, and that is in the possession of a garden or backyard; the absence of this will always be the great drawback to the tenement house. Now although a space for these purposes cannot be provided in a tenement house, still it is possible to provide an open space 20 to 30 square feet to each tenement, on which the w.c. can open, and where a few plants can be grown in boxes or pots; but in well-planned tenement blocks proper spaces must also be made between the blocks which can be used as playgrounds for children, breathing spaces for adults, and drying-grounds for housewives. The tenements should also be so planned that each one stretches from one side of the block to the other, thereby assuring thorough ventilation. Thus the front door with its balcony may be directed, say, towards the east, and the windows of some of the rooms look out on the west side of the block, so that a breeze coming from either of these quarters will sweep right through the tenement, leaving no stagnant corners which cannot be flushed periodically by air at the desire of the occupant. This flushing process can further be facilitated by arranging for windows, fireplaces, and doors to be placed in different sides in each room. Coming to the rooms, each bedroom, sitting-room, and kitchen should have a fireplace—that for the kitchen should be a stove suitable for cooking—the gas stove should be placed in the scullery and never allowed in a living-room or bedroom. Cupboards should be provided for food, fuel, etc., and a copper for boiling clothes should be placed in the scullery, and this brings us to the second part of the subject—viz. the relation of the tenements to one another.

### Relationship of Tenements to Each Other.

The usual plan is for a large space to be mapped out, so that the tenements can be gathered together in oblong-shaped blocks, each something the shape of a domino, with a space between the ends of each block of about 20 feet and a larger space of from 40 to 50 feet or more between the long sides of the block. There is no difficulty in mapping out your blocks as regards superficial area if you adopt the principle that no block should be of greater thickness than a single tenement. Thus there will be no question of "back-to-back" or "side-to-side" tenements, for each tenement will have an outlook on opposite points of the compass. When, however, you come to the question of how many tenements you can place one above the other your difficulties will begin. From a purely commercial standpoint the more tenements you can place on a given space the better it pays, but this is a somewhat short-sighted view, because if the buildings go much over three or four stories high they will be avoided by the better class of tenant, they are difficult to supervise, and are very dangerous in case of fire. The poorer classes dislike these block dwellings and never go into them voluntarily, but as some form of tenement dwellings are a necessity in the central parts of large cities the best course would seem to be to limit their height to three stories, so that by suitable planning the more obvious disabilities of the very high buildings can be avoided, while a moderate rental will secure a sufficient return for the initial outlay.

One of the great difficulties of very high buildings is the collection of household refuse, and by limiting the height as here suggested the dust created can be done away with altogether, and a suitable dustbin with a proper cover can be provided in each tenement, so that the dustman when he calls can carry it downstairs to the dust-cart. In some cases it may be feasible to arrange that the bin be left by the tenant at a certain hour every day in a space provided at the bottom of the common staircase, but this is a detail which can be arranged with the sanitary authority. In this way most of the evils inherent in crowding too many people on a given area will be avoided, and something approaching the home comforts of the cottage dwelling be retained.

